# Establishment and validation of a predictive nomogram for gestational diabetes mellitus during early pregnancy term: A retrospective study

**DOI:** 10.3389/fendo.2023.1087994

**Published:** 2023-02-24

**Authors:** Luman Li, Quan Zhu, Zihan Wang, Yun Tao, Huanyu Liu, Fei Tang, Song-Mei Liu, Yuanzhen Zhang

**Affiliations:** ^1^ Department of Obstetrics and Gynaecology, Zhongnan Hospital of Wuhan University, Wuhan, China; ^2^ Hubei Clinical Research Center for Prenatal Diagnosis and Birth Health, Zhongnan Hospital of Wuhan University, Wuhan, China; ^3^ Hubei Provincial Key Laboratory of Developmentally Originated Diseases, Wuhan University, Wuhan, China; ^4^ Department of Obstetrics, Maternal and Child Health Hospital of Hubei Province, Tongji Medical College, Huazhong University of Science and Technology, Wuhan, China; ^5^ Department of Clinical Laboratory, Center for Gene Diagnosis & Program of Clinical Laboratory Zhongnan Hospital Wuhan University, Wuhan, China

**Keywords:** GDM, nomogram, validation, prediction model, early pregnancy term

## Abstract

**Objective:**

This study aims to develop and evaluate a predictive nomogram for early assessment risk factors of gestational diabetes mellitus (GDM) during early pregnancy term, so as to help early clinical management and intervention.

**Methods:**

A total of 824 pregnant women at Zhongnan Hospital of Wuhan University and Maternal and Child Health Hospital of Hubei Province from 1 February 2020 to 30 April 2020 were enrolled in a retrospective observational study and comprised the training dataset. Routine clinical and laboratory information was collected; we applied least absolute shrinkage and selection operator (LASSO) logistic regression and multivariate ROC risk analysis to determine significant predictors and establish the nomogram, and the early pregnancy files (gestational weeks 12–16, *n* = 392) at the same hospital were collected as a validation dataset. We evaluated the nomogram *via* the receiver operating characteristic (ROC) curve, C-index, calibration curve, and decision curve analysis (DCA).

**Results:**

We conducted LASSO analysis and multivariate regression to establish a GDM nomogram during the early pregnancy term; the five selected risk predictors are as follows: age, blood urea nitrogen (BUN), fibrinogen-to-albumin ratio (FAR), blood urea nitrogen-to-creatinine ratio (BUN/Cr), and blood urea nitrogen-to-albumin ratio (BUN/ALB). The calibration curve and DCA present optimal predictive power. DCA demonstrates that the nomogram could be applied clinically.

**Conclusion:**

An effective nomogram that predicts GDM should be established in order to help clinical management and intervention at the early gestational stage.

## Introduction

Gestational diabetes mellitus (GDM) is a universal metabolic disturbance syndrome with a complicated etiology during pregnancy; insulin resistance and pancreatic *β* cell failure were significant factors for the pathogenesis of the disease, which gradually leads to hyperglycemia (1–4). Hyperglycemia exposure contributes to both maternal and fetal adverse complications. The degree of dysregulation of blood glucose is highly related to the risks of obstetrical and neonatal outcomes, which include cesarean section, hypertension, preeclampsia, polyhydramnios, preterm delivery, fetal growth restriction, birth injury, and respiratory distress. In the long term, there is an increased risk of developing obesity, cardiovascular disease, and type 2 diabetes mellitus in both the mother and the offspring (5). Multiple variables have been reported in previous research, such as age, gestational week, ethnicity, obesity, lifestyle, environment, and metabolism (6, 7). Since the GDM etiology is complicated, the short-term and long-term outcomes are not optimistic and have profound influences, and the demand for early prediction and intervention is increasing.

Two acceptable diagnosis methods that are acknowledged by expert professional organizations such as the International Association of the Diabetes and Pregnancy Study Group (IADPSG) are one-step screening approach (currently preferred by the American Diabetes Association) and the two-step Carpenter–Coustan screening approach (recommended by the American College of Obstetricians and Gynecologists). The one-step screening method can diagnose more patients than the two-step screening method in a large randomized trial, and there is no statistical difference regarding maternal and neonatal adverse outcomes between these two methods (8). Both methods have their own pros and cons, and each has its own cutoff threshold (9). Due to the varying diagnostic criteria, the incidence of GDM varies from 3% to 21.2% in Asia and from 0.31% to 18% globally, and the prevalence continues to rise (10–12). The WHO recommended a 75-g anhydrous glucose load screening test for diagnosis after 8–14 h overnight fasting at 24–28 gestational weeks (13). Because pregnant women undergo the oral glucose tolerance test (OGTT) at the second stage of the trimester, early warning signs for dysglycemia may be missed.

Our study aims at establishing a nomogram to predict the risk factors of GDM during early pregnancy term and to apply early intervention. Early management and intervention of GDM improves maternal and perinatal outcomes (14, 15). Prediction models can correctly identify GDM at early gestational weeks and could mostly benefit women with targeted risk factors, which helps them focus on precision lifestyle changes. These models can be used as tools to identify risk factors and stratify diseases, which can be largely applied in clinical management and treatment (16). Using statistical modeling combined with clinical variables and laboratory information, we developed prediction tools for GDM, which can be applied in early gestational weeks.

## Materials and methods

### Data collection

**Figure 1:**
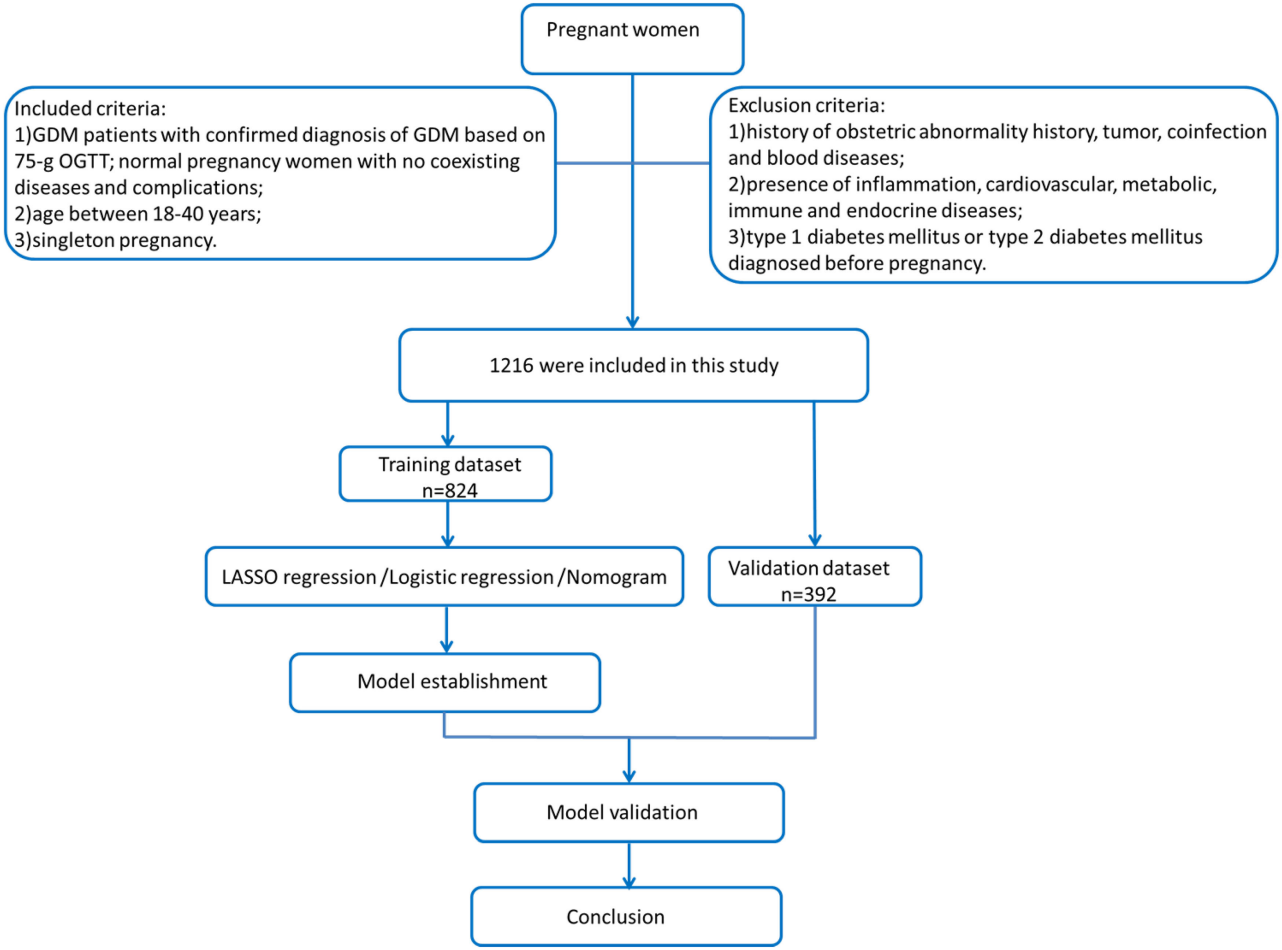
Flowchart of this study. A total of 1,216 pregnant women enrolled in our study were selected by inclusion criteria. The training dataset (*n* = 824) was used to estimate a GDM predictive model, and our study applied LASSO logistic regression and multivariate ROC risk analysis to determine significant predictors and establish the nomogram. A total of 392 early pregnant women were used as a validation dataset. We evaluate the nomogram by AUC, C-index, calibration curve, and decision curve analysis (DCA).

This study is a retrospective study that recruited 1,216 pregnant women at Zhongnan Hospital of Wuhan University and Maternal and Child Health Hospital of Hubei Province from 1 February to 30 April 2020. A total of 824 pregnant women in the second and third trimesters were enrolled in the training dataset, and their clinical and laboratory data during their 12th–16th gestational weeks were retrospectively collected. We also recruited 392 pregnant women during early pregnancy as a validation dataset. We collected the following maternal clinical and laboratory information: age, gestational week, gravidity and parity history, white blood cell (WBC), red blood cell (RBC), platelet (PLT), prothrombin time (PT), activated partial thromboplastin time (APTT), thrombin time (TT), fibrinogen (FIB), total protein (TP), albumin (ALB), alkaline phosphatase (ALP), blood urea nitrogen (BUN), creatinine (Cr), uric acid (UA), fibrinogen-to-albumin ratio (FAR), blood urea nitrogen-to-creatinine ratio (BUN/Cr), and blood urea nitrogen-to-albumin (BUN/ALB). All blood samples were collected by skilled nurses, and the blood tests were taken in the laboratory of Zhongnan Hospital of Wuhan University and Maternal and Child Health Hospital of Hubei Province. The levels of these factors were measured by commercial diagnostic kits: RBC, PLT, and PLT (DXH800, UniCel automated hematology analyzer, USA); PT, APTT, and FIB (CA1500, Sysmex coagulation analyzer, USA); and TT, TP, ALP, ALB, BUN, Crea, and UA (AU5800, Beckman biochemical analyzer, USA). Inclusion criteria were as follows: (1) GDM patients with confirmed diagnosis of GDM based on the 75-g OGTT test (2010 IADPSG criteria (17); cutoff threshold values: 0 h fasting plasma glucose ≥ 5.1 mmol/L, 1 h plasma glucose ≥ 10.0 mmol/L, and 2 h plasma glucose ≥ 8.5 mmol/L) and normal pregnant women with no coexisting diseases and complications; (2) singleton pregnancy; and (3) age between 18 and 45 years. Exclusion criteria were as follows: (1) history of obstetric abnormality history, tumor, coinfection, and blood diseases; (2) presence of inflammation, cardiovascular, metabolic, immune, and endocrine diseases; and (3) type 1 diabetes mellitus or type 2 diabetes mellitus which were diagnosed before pregnancy. The details of our study process are depicted in the flowchart in **Figure 1**. LASSO logistic regression and multivariate ROC risk analysis were applied to establish significant factors and establish the nomogram. The early pregnancy files (gestational weeks 12–16, *n* = 392) were collected as a validation dataset. AUC, C-index, calibration curve, and DCA were used to evaluate the nomogram. The risk factor “age”, which acts as a continuous variable, has a poor predictive value; according to multivariate logistic regression and clinical meaning, we select the cutoff value of “30” to divide “age” as a categorical variable.

### Statistical methods

Statistical analysis was performed with SPSS 24.0 and R 4.0.0 software (R Statistical Computing Foundation, Vienna, Austria). Continuous data were expressed as mean ± standard deviation. Clinical characteristics were compared using *t*-test (continuous variables) and *χ* ^2^ test (categorical variables). LASSO regression was used to select the best predictive factors (18). The nomogram was established as a result of the binary logistic regression model with fivefold cross-validation. Selected factors applied in the nomogram fit the following: selected by multivariable analysis and clinically relevant. The calibration curve was applied to assess the accuracy of the predictive model (the Hosmer–Lemeshow test was used to access goodness of fit). The ROC curve evaluates discriminative ability by the area under the ROC curve (AUC). The DCA curve was conducted to determine the clinical utility and benefit of the nomogram. All cutoff values were determined by the total risk scores in the training cohort. Differences with *p*-value < 0.05 were considered statistically significant.

Based on the 10EPP rule (19), the sample size of our predictor model should be at least 170; our study sample consists of 824 women in the training dataset and 392 women in the validation dataset, and based on the sample size of our study, the power (1 − *β*) calculation is equal to 1.0.

## Results

### Patients’ clinical characteristics

We included 824 pregnant women in the training cohort and 392 pregnant women in the validation dataset. All the *p*-values of these factors are greater than 0.001, which indicates that there were no statistically significant differences between the training dataset and the validation dataset as shown in **Table 1**. The baseline characteristics of each dataset are presented in **Table 2**, in which data on non-GDM and GDM pregnant women from both datasets are shown separately. We selected the following predictive factors by logistic regression analysis: age, WBC, PLT, APTT, BUN, UA, FAR, BUN/Cr, and BUN/ALB. Then, we selected the statistically significant factors in multivariate logistic regression and clinical correlated factors to establish a predictive model, including the following factors: age, FAR, BUN, BUN/Cr, and BUN/ALB (shown in **Table 3**).

**Table 1:** A summary of the variables grouped by training and validation dataset in this study.

Variable	Training dataset (n=824)	Validation dataset (n=392)	*p*
Age (years)	30±4.1	30±3.3	0.11
Gestational weeks	14±1.7	14±1.2	0.82
Laboratory results			
RBC (10^12/L)	4.0±1.2	4.0±0.4	0.96
WBC (10^9/L)	9.8±3.0	9.8±3.0	0.85
PLT (10^9/L)	217±89.7	218.2±56.9	0.89
PT (s)	10.5±3.5	10.4±0.7	0.45
APTT (s)	26.6±2.3	26.6±2.4	0.99
TT (s)	12.6±1.7	12.4±1.7	0.03
TP (g/L)	66.4±5.3	66.5±4.7	0.47
ALP (U/L)	204.5±108.1	205.2±104.2	0.39
FIB (mg/dL)	443.0±62.6	443.4±58.7	0.69
ALB (g/L)	35.4±3.5	35.4±3.5	0.87
BUN (mmol/L)	3.4±1.0	3.4±1.1	0.10
Crea (μmol/L)	49.8±9.0	50.3±9.3	0.15
UA (μmol/L)	318.0±79.0	319.9±79.2	0.29
FAR	12.6±2.2	12.7±2.2	0.83
BUN/Cr ratio	6.9±1.9	7.0±1.9	0.06
BUN/ALB ratio	9.7±3.3	10.0±3.7	0.12

Bold value means *p*-value < 0.05, which indicates statistically significant.

**Table 2:** The baseline characteristics of datasets.

Variables	Training dataset			Validation dataset		
	Non-GDM	GDM	*p*	Non-GDM	GDM	*p*
n	620 (75.2)	204 (24.8)		196	196	
Age (years)			** 0.01**			**0.03**
18-30	532 (63.5)	158 (19.2)		157 (80.1)	157 (80.1)	
≥30	87 (10.6)	46 (5.6)		39 (18.9)	39 (18.9)	
Laboratory results						
RBC (10^12/L)	4.0±0.5	4.0±1.3	0.87	4.0±0.4	4.0±0.4	0.82
WBC (10^9/L)	9.4±3.0	10.0±3.0	**0.040**	10.2±2.9	9.4±3.0	**0.010**
PLT (10^9/L)	205.8±59.6	221.8±97.6	**0.031**	230.5±50.8	206.7±59.7	**<0.001**
PT (s)	10.4±0.5	10.6±4.0	0.42	10.5±0.9	10.4±0.5	0.16
APTT (s)	26.9±2.4	26.5±2.3	**0.042**	26.3±2.4	26.9±2.4	**0.037**
TT (s)	12.8±1.9	12.6±2.3	0.080	12.0±1.3	12.8±1.9	**<0.001**
TP (g/L)	66.1±5.4	66.5±5.3	0.29	67.0±3.9	66.1±5.4	0.079
ALP (U/L)	202.0±117.5	205.3±104.9	0.71	206.5±87.6	203.4±118.8	0.80
FIB (mg/dL)	439.2±68.2	449.9±59.1	0.054	438.4±58.5	448.3±58.8	0.085
ALB (g/L)	35.4±3.3	35.2±4.0	0.39	35.5±2.8	35.2±4.0	0.36
BUN (mmol/L)	3.3±0.9	3.7±1.2	**<0.001**	3.3±1.0	3.7±1.2	**<0.001**
Crea (μmol/L)	49.4±8.5	50.9±10.0	**0.039**	49.7±8.6	50.8±10.1	0.27
UA (μmol/L)	313.7±76.5	330.8±84.9	**0.007**	308.4±71.8	331.4±84.5	**<0.001**
FAR	12.5±2.3	12.9±2.3	**0.025**	12.4±2.1	12.9±2.3	**0.025**
BUN/Cr ratio	6.7±1.8	7.4±2.0	**<0.001**	6.6±1.7	7.4±2.0	**<0.001**
BUN/ALB ratio	9.3±3.1	10.7±3.9	**<0.001**	9.4±3.5	10.7±3.8	**<0.001**

Bold value means *p*-value < 0.05, which indicates statistically significant.

**Table 3:** Multivariable logistic model of probability of GDM in training dataset.

Variables	OR	B	*p*	95%CI	
Age (years)	1.93	0.66	**<0.001**	1.34	2.79
BUN (mmol/L)	1.59	0.46	**<0.001**	1.33	1.91
FAR	1.61	0.47	**0.017**	1.09	2.37
BUN/Cr ratio	2.38	0.87	**<0.001**	1.66	3.40
BUN/ALB ratio	2.31	0.84	**<0.001**	1.61	3.33

Bold value means *p*-value < 0.05, which indicates statistically significant.

### Development and validation of the nomogram

**Figure 2:**
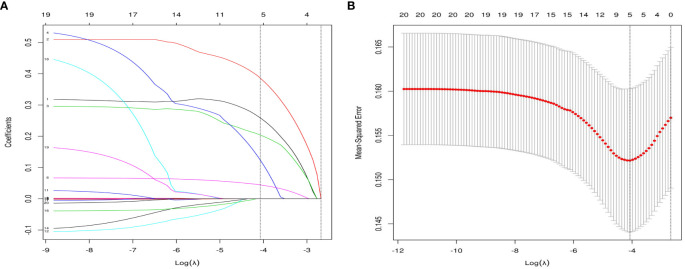
Variable selection by the LASSO binary logistic regression model. **(A)** eighteen variables with nonzero coefficients were selected by deriving the optimal lambda. **(B)** Following verification of the optimal parameter (λ) in the LASSO model, the mean squared error changes with respect to the Log (λ) value, and the vertical dotted line near Log (λ) = −4 is drawn based on 1 standard error criteria.

**Figure 3:**
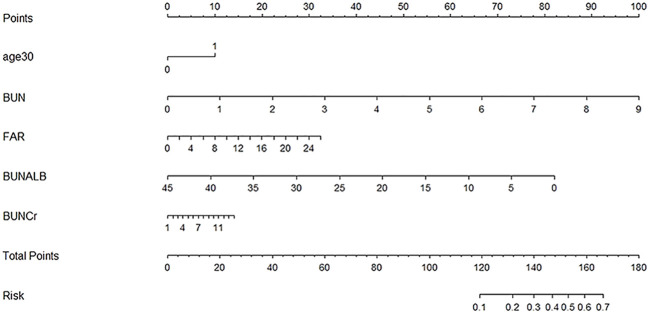
Nomogram to estimate the probability of GDM. A nomogram used basic pregnancy file information to predict GDM. Find the predictor points on the uppermost point scale that correspond to each variable of the pregnant woman and add them up; the total points projected to the bottom scale indicate the probability of GDM.

**Figure 4:**
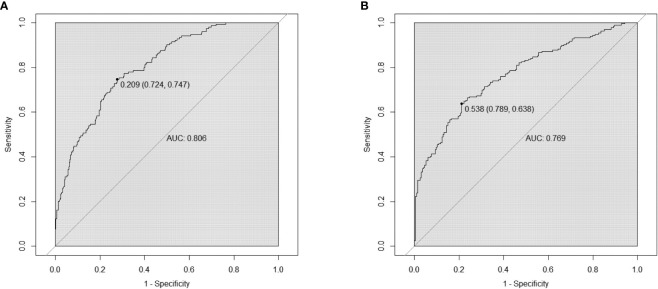
Receiver operating characteristic (ROC) curves of nomograms in the training dataset and validation dataset, respectively. **(A)** The AUC value of the training dataset is 0.808, 95% CI: 0.770–0.842 (*p* < 0.05). **(B)** The AUC value of the validation dataset is 0.769, 95% CI: 0.722–0.815 (*p* < 0.05).

**Figure 5:**
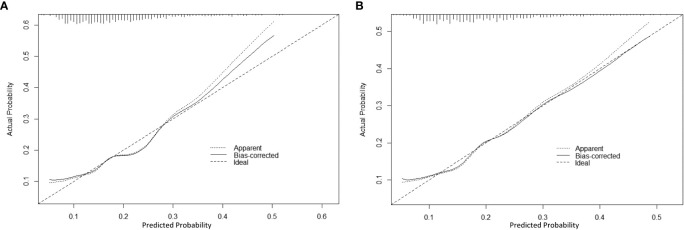
The calibration curve of the nomogram for predicting GDM in the training dataset and validation dataset, respectively. Calibration focused on the accuracy of the probability between the predictive model and the actually observed value. The *y*-axis represents the actual diagnosed cases of GDM, the *x*-axis represents the predicted risk of GDM, and the solid line represents the prediction of the training dataset **(A)** and the validation dataset **(B)**.

**Figure 6:**
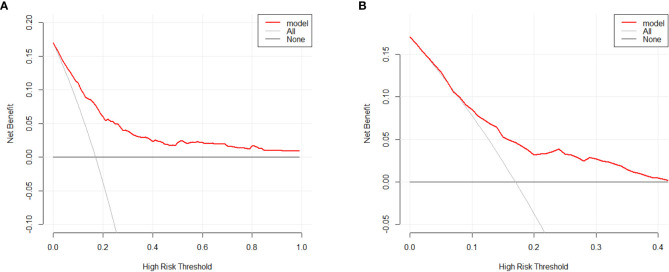
Decision curve analysis for the GDM risk nomogram. The *y*-axis estimates the net benefit, the transverse solid line represents the probability of risk that pregnant Asian women have no GDM, and the oblique solid line represents the probability of risk that pregnant Asian women have GDM. **(A)** Training dataset. **(B)** Validation dataset.

Based on the factors selected from the training cohort, LASSO regression analysis was conducted to select the predictive factors from **Table 1** and establish the model with factors shown in **Table 2**: Five of the eighteen variables were enrolled to build the predictive model (**Figure 2**). These selected factors showed significant statistical differences, and they were independent of each other. The “Rms” package was used to build a nomogram to establish a GDM diagnosis model; the nomogram was constructed to predict the risk of GDM during early pregnancy (**Figure 3**). These five variables are given in **Table 3**. The AUC aimed to evaluate the discrimination of the nomogram in **Figure 4**; the AUC value of the training dataset is 0.808, 95% CI: 0.770–0.842 (*p* < 0.05, **Figure 4A**), and the AUC value of the validation dataset is 0.769, 95% CI: 0.722–0.815 (*p* < 0.05, **Figure 4B**). The calibration curve was used to evaluate the predictive power shown in **Figure 5**. The predictive model and the validation set showed the optimal predictive degree of the fitting. The DCA demonstrated the threshold probability of the prediction model nomogram in the training and validation datasets, respectively, and it was used to evaluate the clinical effects of the nomogram more visually, which indicated that the nomogram has optimal predictive power. DCA demonstrated that the nomogram could be applied clinically (**Figure 6**).

## Discussion

GDM is defined as dysglycemia with onset or first recognition during pregnancy (20); insulin resistance and pancreatic *β* cell failure have been reported to be significant factors in GDM aside from the other main causes of GDM such as maternal age, obesity, inflammation, and inadequate physical exercise (21). GDM increases maternal and neonatal adverse effects in both short-term and long-term periods. In addition, it is necessary to identify and address the risk factors of GDM early and accurately. Tools that could accurately target these GDM predictors in early pregnant women will most likely benefit these women (22).

On the other hand, early warning and intervention during early pregnancy may prevent the adverse outcomes of GDM by controlling glucose level. The first-line treatment for GDM is medical nutrition therapy, weight management, and physical activities (23–25). to "70% to 85% of women diagnosed with GDM could modify their glucose condition through targeted lifestyle changes (26). In general, an early prediction model of GDM should be established, which could positively affect prevention, treatment, and prognosis.

Prior studies indicated that BUN was dose-response related with GDM during the first trimester (27). Diabetes mellitus drives the occurrence of kidney diseases (28). Meanwhile, kidney metabolites such as urea or other uremic components may increase the risk of diabetes (29). BUN was considered as a kidney function marker; a high level of urea increases insulin resistance and suppresses insulin secretion, which is associated with an increased risk of incident diabetes mellitus (30). The underlying mechanism is as follows: urea induced the production of reactive oxygen species and restrains insulin signaling by suppressing insulin receptor substrate–serine phosphorylation (31); on the other hand, uremic metabolite accumulation impaired *β*-cell normal function and negatively affected glucose homeostasis (32).

Meanwhile, fibrinogen is a long-acting plasma acute-phase reactant (33), and the change in albumin level has been attributed to the changes in nutritional status; furthermore, hypoalbuminemia represents a chronic inflammatory state caused by malnutrition (33, 34). Likewise, FAR has been proven to be a more powerful inflammatory-based prognostic predictor of overall survival than other single prognostic markers (35–37); compared with healthy pregnancies, FAR was considered to be an independent risk factor for predicting spontaneous abortion, and increased FAR levels were considered to be related to the thrombotic process in recurrent abortion (38, 39). BUN/Cr is an important indicator to evaluate acute renal injury and gastrointestinal hemorrhage, and a low BUN/Cr level is associated with higher risks of total and ischemic stroke (40–42). BUN/ALB is a novel prognostic marker that has a higher predictive ability than single urea nitrogen and albumin in pneumonia and acute pulmonary embolism (43–46). Given the low cost and the abundance of laboratory offerings, and the fact that these markers provided poor clinical outcomes in previous studies, we generated a predictive nomogram of GDM through serial measures.

From an economics perspective, our study takes advantage of early pregnancy files and validates the nomogram that was set up for 824 enrolled pregnant women. In this multicenter study, we have identified five predictors, namely, age, BUN, FAR, BUN/ALB, and BUN/Cr, which were significantly associated with GDM. These five predictors are independent of each other, and research about their relationship has rarely been reported. We also developed a nomogram that could predict the incidence of GDM during early pregnancy.

Our study has strengths and limitations; this is a multicenter retrospective study with a large sample size of pregnant women, and we used an early pregnant stage dataset verified by the nomogram. The GDM predictive nomogram focused on several clinical factors which could be readily available at low cost via routine blood tests in clinical practice, and the nomogram can be performed with optimal predictive power with better combined clinical characteristics with laboratory results. This model can be widely used in less-developed and developing countries where the incidence of GDM is rapidly increasing. It provides risk assessment based on first pregnancy profiles for early detection and intervention and to control glucose level. Thus, it should be widely carried out in more basic-level hospitals. However, many factors should be considered first. We should expand the sample size *via* dynamic monitoring of different gestational weeks and detect more variables and risk factors during pregnancy before the model can be widely used in clinical practice.

In summary, by analyzing basic information from pregnancy files, we found five independent risk factors of GDM: age, BUN, FAR, BUN/Cr, and BUN/ALB. According to the GDM nomogram predictive model validated by the early pregnancy dataset, we could help patients’ clinical management at the early gestational stage.

## Data availability statement

The datasets presented in this article are not readily available due to the nature of this research, participants of this study did not agree for their data to be shared publicly, so supporting data is not available. Requests to access the datasets should be directed to zhangyuanzhen@whu.edu.cn.

## Ethics statement

The study was approved by the Institutional Ethics Committee of the Zhongnan Hospital of Wuhan University and the Maternal and Child Health Hospital of Hubei Province, the informed consent number was No. 2020072K.

## Author contributions

LL wrote the first draft of the manuscript. LL, S-ML, and YZ contributed to the conception and design of the study. YZ is the first corresponding author. QZ, ZW, and FT collected the data. LL, QZ, and HL performed the data processing and analysis. YT contributed to the critical revision of the manuscript. All authors contributed to manuscript revision, and read and approved the submitted version.
